# Supplementary material for: The Lotus japonicus alpha‐expansin EXPA1 is recruited during intracellular and intercellular rhizobial colonization
Source: Plant J. 2025 Dec 11;124(5):e70639. doi: 10.1111/tpj.70639 (PMC13020659; doi:10.1111/tpj.70639)
Supplement: Supplementary file 3 — Figure S1. Variable expression pattern of Lotus Expansins in different organs and tissues. Figure S2. Phylogeny and composition of the Expansin family in different plant species. Figure S3. Amino acid sequence alignment of LjEXPA1 with Arabidopsis and Bacillus subtillis expansins. Figure S4. Subcellular localization of LjEXPA1 at the transcellular passage cleft (TPC). Figure S5. Subcellular localization of LjEXPA1 during nodule primordia formation. Figure S6. LORE1 insertions and plant growth phenotype in expA1 mutants. [file TPJ-124-e70639-s003.pdf]

Supporting Information

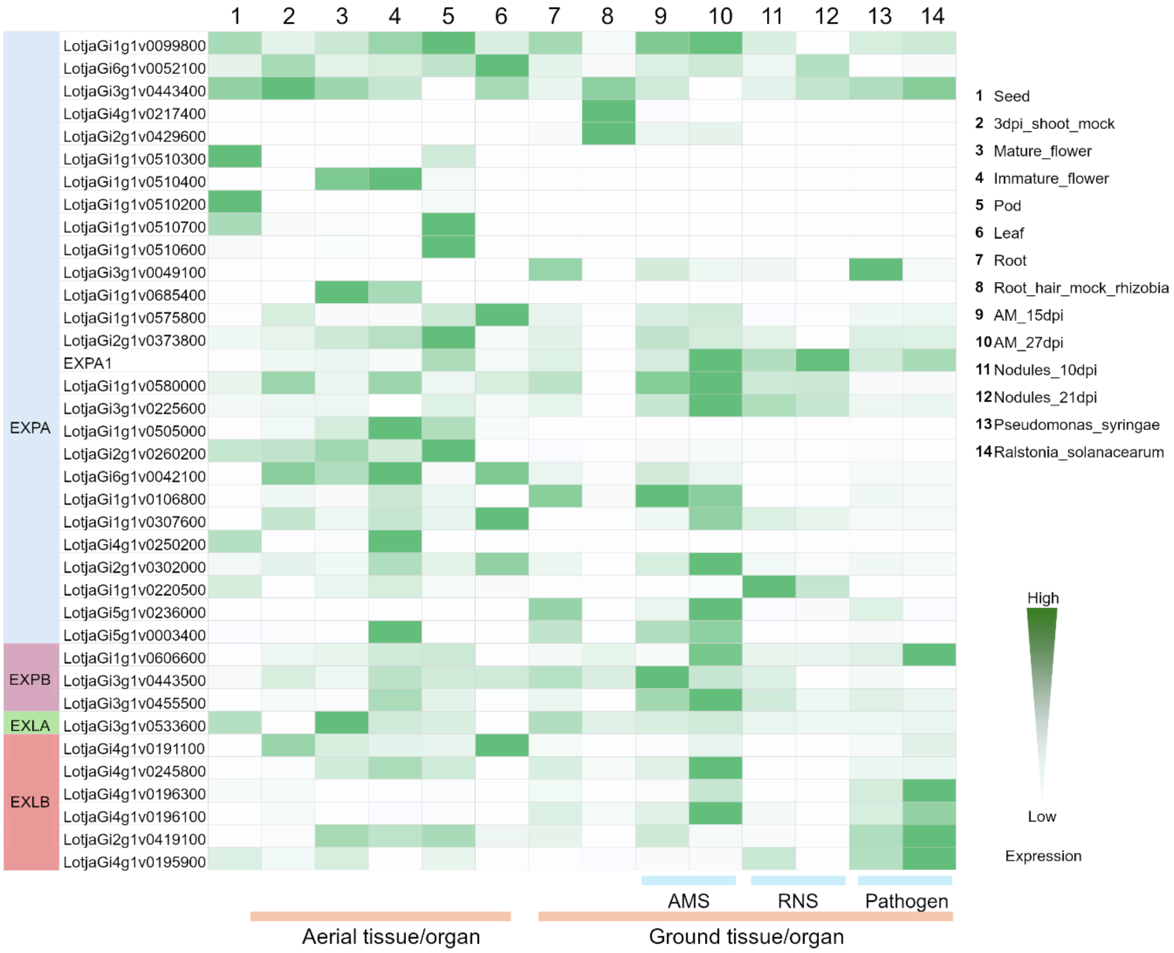

Figure S1. Variable expression pattern of *Lotus Expansins* in different organs and tissues. Heat map gene expression of *Lotus Expansins* in aerial and ground tissues/organs, under non-symbiotic, symbiotic and pathogenic conditions. Data collected from *Lotus* base (https://lotus.au.dk/). AMS, arbuscular mycorrhiza symbiosis; RNS, root nodule symbiosis.

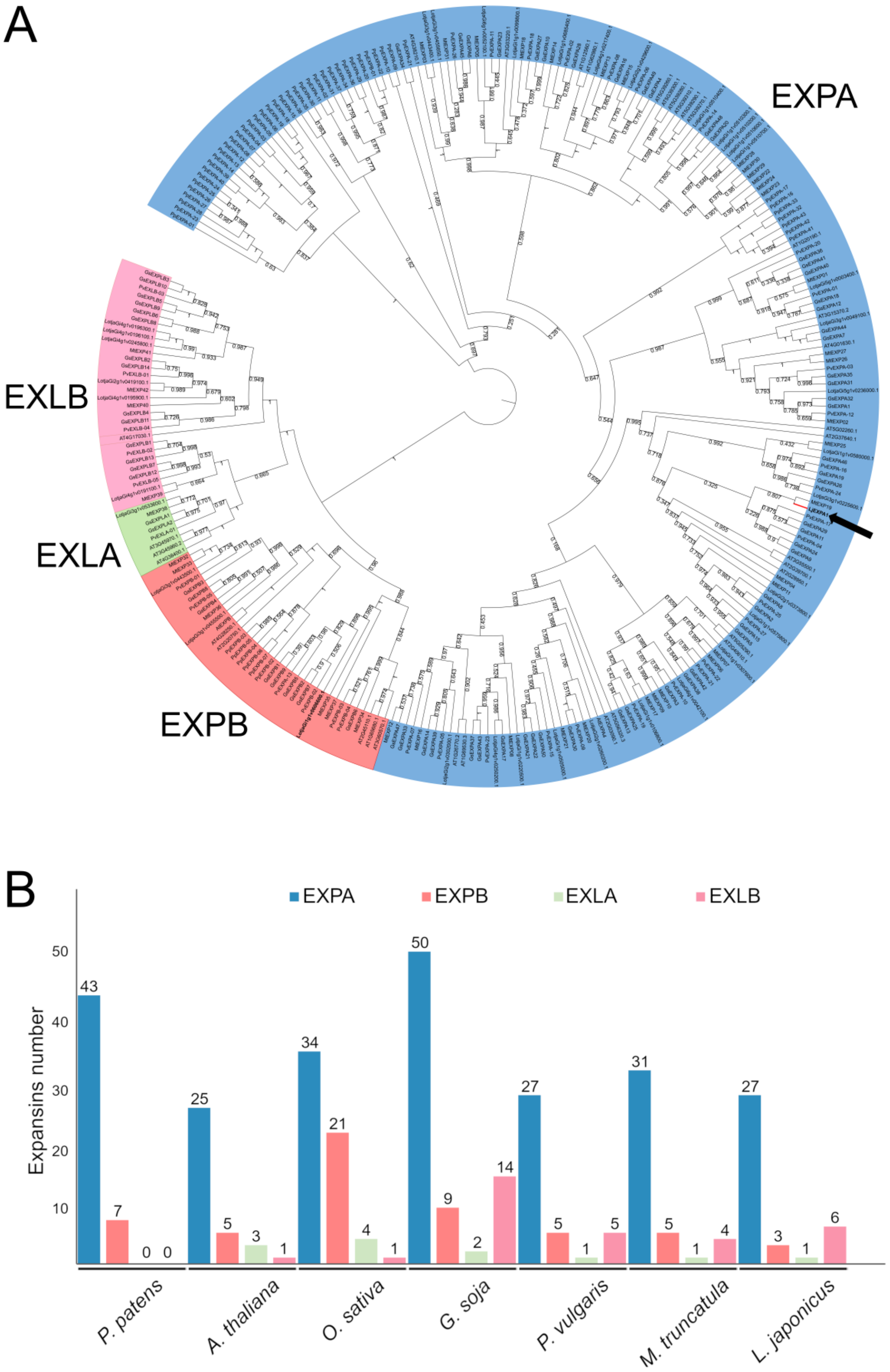
A
EXPA
EXLB
EXLA
EXPB
B
EXPA
EXPB
EXLA
EXLB
Expansins number
50
40
30
20
10
43
7
0
0
25
5
3
1
34
21
4
1
50
9
2
14
27
5
1
5
31
5
1
4
27
3
1
6
P. patens
A. thaliana
O. sativa
G. soja
P. vulgaris
M. truncatula
L. japonicus

Figure S2. Phylogeny and composition of the Expansin family in different plant species. Maximum likelihood phylogenetic tree **(A)** and number **(B)** of alpha, beta, alpha-like and beta-like Expansins in *Physcomitrella patens, A. thaliana, Oryza sativa, Glycine soja, Phaseolus vulgaris, Medicago truncatula* and *L. japonicus*. EXPA1 is highlighted with an arrow. LotjaGi1g1v060600, marked with an asterisk, is the putative orthologue of GmEXPB2 (Li et al. 2015). Bootstrap values are shown in the branches (1000 bootstrap replicates).

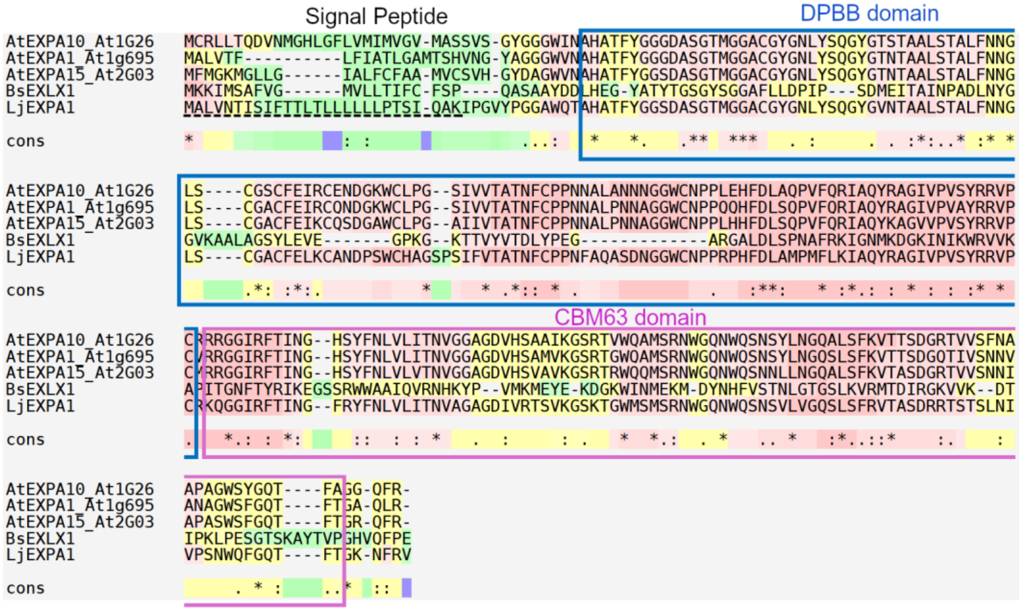

Figure S3. Amino acid sequence alignment of LjEXPA1 with *Arabidopsis* and *Bacillus subtillis* Expansins. LjEXPA1 contains the two characteristic domains of alpha expansins: a DPPB motif at the N-terminus and the carbohydrate-binding region known as CBM63. Notably, it features a conserved aspartic acid residue (indicated by an arrow), which is essential for cell wall loosening as demonstrated in the *B. subtilis* Expansin EXLX1 (Georgelis *et al.*, 2011). The signal peptide is marked with a black dashed line.

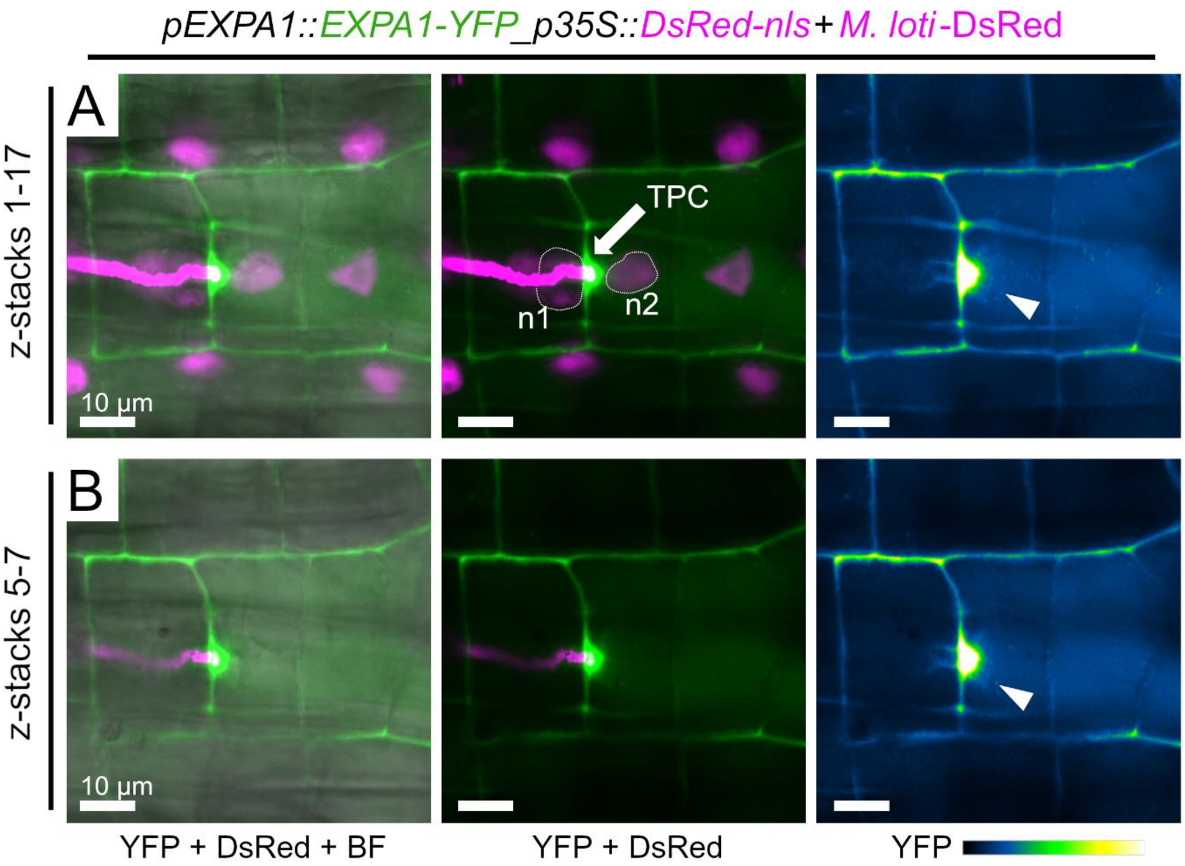

Figure S4. Subcellular localization of LjEXPA1 at the transcellular passage cleft (TPC). Live cell confocal images showing a close-up of the region outlined by the dashed white line box in Figure 3C. A strong accumulation of EXPA1 is visible at the TCP (arrow), alongside with a weaker labelling of small intracellular punctate structures (arrowhead) surrounding the site, possibly representing secretion compartments. A white dotted freehand line encircles both the nucleus of the cell hosting the progressing IT (n1) and the nucleus of the recipient cell (n2). Images are maximum intensity projections of 17 (**A**) or 3 (**B**) stacks from the same z-stack, showing the merge of the YFP (green) and DsRed (magenta) channels with (left) or without (right) the bright field (BF) channel. See Movie 1 and 2.

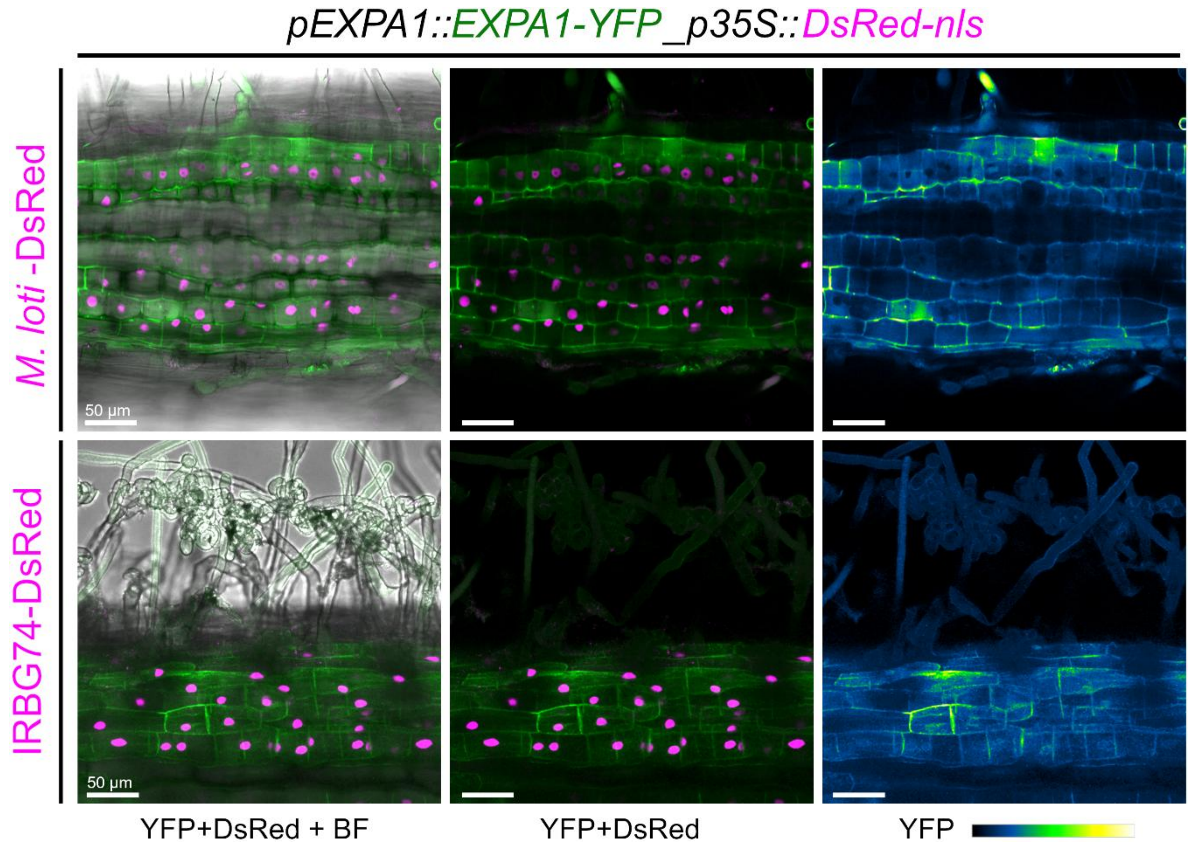

Figure S5. Subcellular localization of LjEXPA1 during nodule primordia formation. Live-cell confocal images of *Lotus* transgenic roots expressing the *pEXPA1::EXPA1-YFP_p35S: :DsRed-nls* construct during nodule primordia formation after *M. loti* R7A-DsRed **(A)** and IRBG74-DsRed colonization **(B)**. Images are single focal planes **(A)** or maximum intensity projections of z-stacks (**B**) showing either the merge of the YFP (green) and DsRed (magenta) channels with (left) or without (middle) the bright field (BF) channel, or the isolated YFP channel (green fire blue, right). At least twelve composite plants from two independent experiments were analysed.

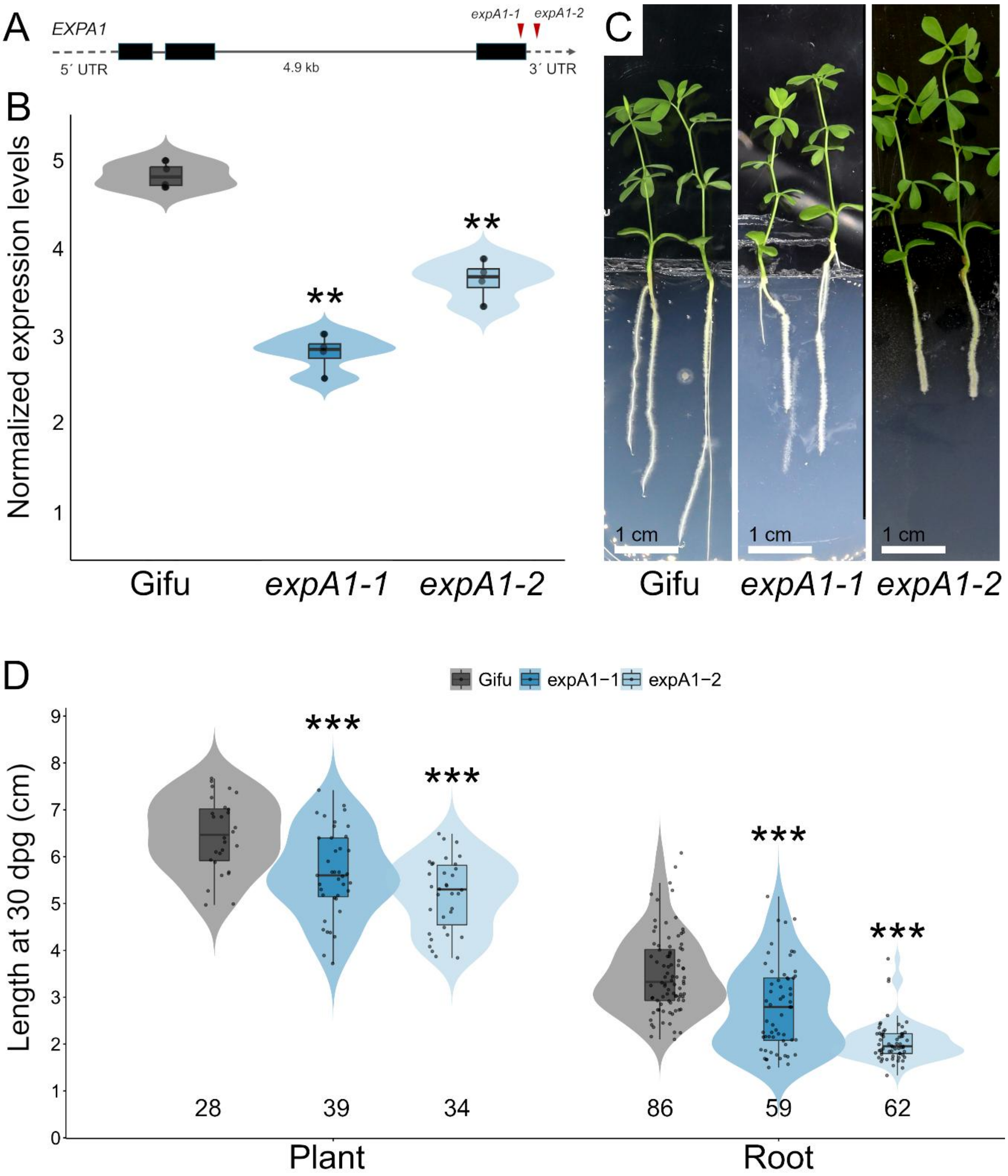

Figure S6. *LORE1* insertions and plant growth phenotype in *expA1* mutants. A, Schematic representation of *LjEXPA1* gene disrupted by LORE1 insertions. Black boxes: exons; Dashed lines, UTRs; Solid lines, introns. Retrotransposon insertions are indicated with red triangles. **B,** Boxplots represent the normalized expression levels of *EXPA1,* calculated by RT-qPCR from 4 independent biological replicates (n = 5 plants per biological replicate) in Gifu, *expa1*-1 and *expA1*-2 roots. **C,**

Representative images of Gifu, *expa1*-1 and *expA1*-2 grown in square Petri dishes with nitrogen-repleted medium at 30 dpg. **D,** Plant and root length at 30 dpg. In boxplots, the center line represents means values of 3 independent experiments; box limits, upper and lower quartiles; whiskers, 1.5× interquartile range; points represent individual data points. The asterisk indicates statistical significance between the *LORE1* mutant and Gifu according to Student´s *t* test (****P* < 0.001).
